# False-positive Imaging for Papillary Thyroid Cancer Caused by Intraosseous Hemangiomas

**DOI:** 10.1210/jcemcr/luad102

**Published:** 2023-09-27

**Authors:** Heejoo Kang, Frederick Thurston Drake, David McAneny, Stephanie L Lee

**Affiliations:** Department of Surgery, Section of Endocrine Surgery, Chobanian & Avedisian School of Medicine, Boston University, Boston, MA 02118, USA; Department of Surgery, Section of Endocrine Surgery, Chobanian & Avedisian School of Medicine, Boston University, Boston, MA 02118, USA; Department of Surgery, Section of Endocrine Surgery, Chobanian & Avedisian School of Medicine, Boston University, Boston, MA 02118, USA; Department of Medicine, Section of Endocrinology, Nutrition, Diabetes, and Weight Management, Chobanian & Avedisian School of Medicine, Boston University, Boston, MA 02118, USA

**Keywords:** false-positive scan, thyroid cancer, intraosseous hemangioma, whole body scan, CT scan, MRI scan, lytic bone

## Abstract

Two patients with papillary thyroid carcinoma and an elevated thyroglobulin had false-positive imaging studies from intraosseous hemangiomas (IH). A 62-year-old man presented with a palpable lytic skull mass suspicious for a bone metastasis after computed tomography (CT) and magnetic resonance imaging (MRI) scans. Surgical excision confirmed an IH. The second patient is a 64-year-old woman whose I-123 whole-body scan with single photon emission computed tomography/CT demonstrated radioiodine uptake in the right frontal bone. Her MRI and CT scans were also consistent with an IH. These cases reveal the limitations of nuclear imaging and of CT and MRI scans in distinguishing metastatic differentiated thyroid cancer from IH in patients with lytic bone lesions. Because no imaging studies are definitive for an IH, bone cranial lesions may warrant resection to establish a diagnosis and avoid potential brain invasion by a malignancy or unnecessary radioiodine treatment.

## Introduction

Thyroid cancer is the most common endocrine carcinoma [1]. The evaluation of intermediate- and high-risk patients with thyroid cancer may involve cross-sectional imaging including radioiodine whole body scan (WBS) with single photon emission computed tomography (SPECT) imaging, computed tomography (CT), magnetic resonance imaging (MRI), and/or positron emission tomography (PET)/CT scans [1]. All imaging scans are associated with false-positive results when assessing for metastatic thyroid cancer [2, 3] and rarely caused by intraosseous hemangiomas (IH) [2, 4]. Radioactive iodine uptake is expected in thyroid remnants and metastases, but retention of the isotope has also been described in cystic lesions of the skin, gallbladder, and kidneys [2, 3]. Lytic bone lesions are often associated with metastatic carcinomas, especially thyroid cancer, but they may also occur in benign conditions such as bone cysts, fibrous dysplasia, giant cell tumors, and brown tumors of hyperparathyroidism. This report demonstrates that IH can also mimic thyroid cancer metastases in lytic bone lesions.

## Case Presentations

### Case 1

**Figure 1.**
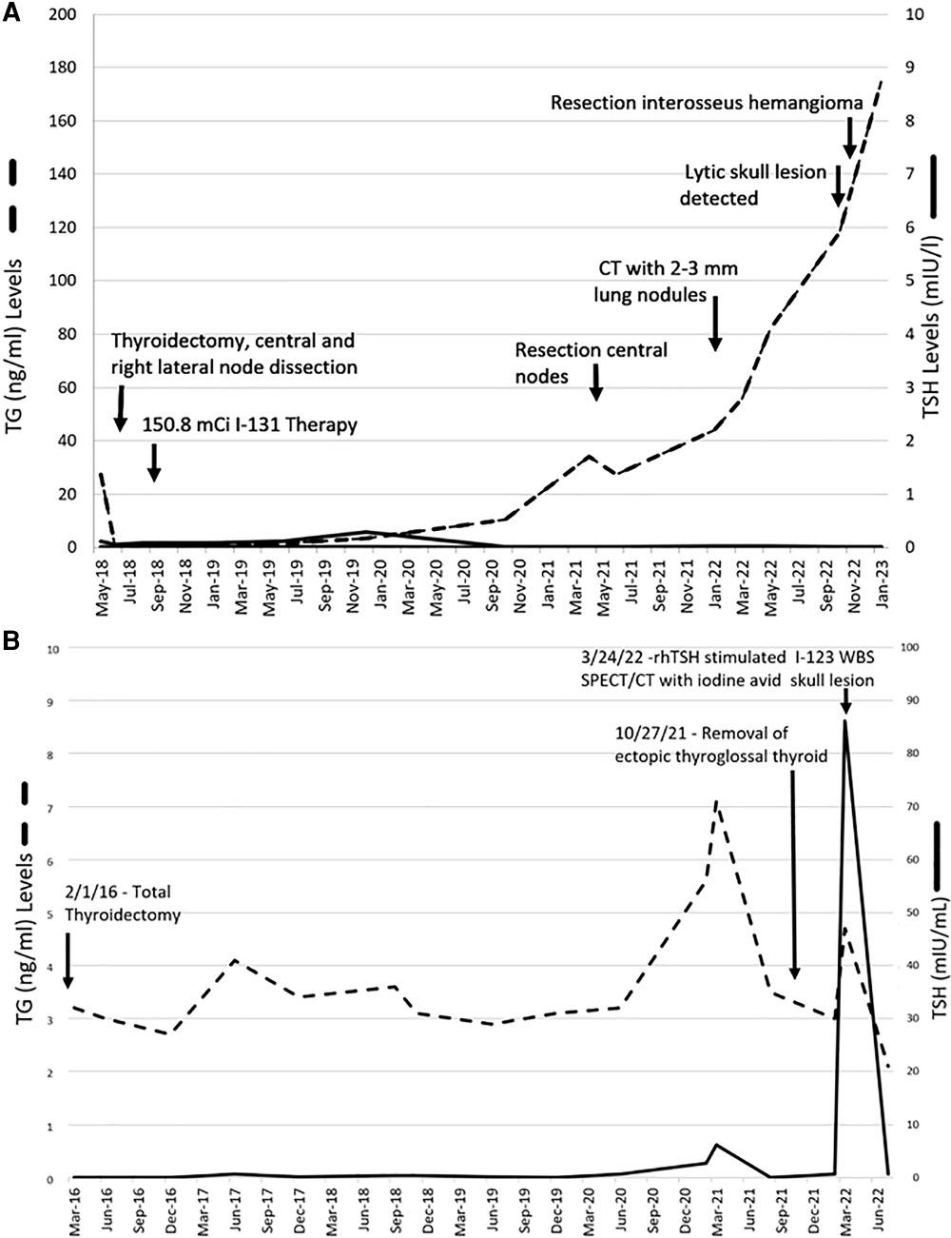
Thyroglobulin trends for high-risk case 1 (A) and low-risk case 2 (B) patients with papillary thyroid cancer, elevated thyroglobulin levels, and lytic bone lesions in the cranium.

A 62-year-old man had a thyroidectomy and node dissection for a 5-cm columnar cell variant of papillary thyroid cancer (PTC) with minimal extrathyroidal extension, extensive vascular invasion, and lymphatic invasion. Nine of 35 nodes (<1 cm) contained metastatic PTC with extranodal extension. He was treated with 150.8 mCi of radioactive iodine (131-I). The posttherapy SPECT/CT scans revealed only residual thyroid tissue, with no suspicious lung nodules. His posttreatment thyroglobulin (Tg) level was 0.34 ng/mL (0.34 µg/L) with a negative Tg antibody < 0.4 U/mL (<0.4 kIU/L), and a neck ultrasound was unremarkable. The original tumor staging was American Joint Committee on Cancer 8th edition stage II and the American Thyroid Association risk stratification was intermediate with an indeterminate response to therapy. One year later, a recombinant human TSH-stimulated I-123 WBS demonstrated no radioiodine uptake, but the stimulated Tg rose to 2.2 ng/mL (2.2 µg/L). The patient was not reevaluated until 3 years after diagnosis, when the TSH-suppressed Tg level rose to 34.1 ng/mL (34.1 µg/L) (Fig. 1A), and ultrasonography showed 3 foci of recurrent tumor (1-cm superficial mass in the right thyroid bed, 1.3-cm mass superficial to the cricoid cartilage, and 0.5-cm mass in a right level II lymph node). A CT scan of the neck and chest and a PET/CT scan revealed hypermetabolic masses concordant with ultrasonography with no evidence of distant disease. However, none of these studies included the skull superior to the orbital ridge. A reoperative dissection of the right central and lateral compartments removed all gross disease, although his Tg level remained elevated at 27.3 ng/mL (27.3 µg/L) and continued to rise to 44.0 ng/mL (44.0 µg/L) (Fig. 1A). His chest CT scan demonstrated multiple new 2- to 3-mm lung nodules, consistent with metastatic cancer. Two months later, the patient noticed a 1.5-cm tender skull mass, when his TSH-suppressed Tg was 118.0 ng/mL (118.0 µg/L). His examination revealed a sensitive, hard, fixed skull mass.

### Case 2

A 64-year-old woman underwent a thyroidectomy for a large nodular goiter that contained a 2.1-cm encapsulated follicular variant of PTC with focal capsular invasion without lymphovascular invasion or extrathyroidal extension. This tumor was regarded as American Joint Committee on Cancer stage I and American Thyroid Association low risk for recurrence, but the unstimulated Tg rose from 2.7 to 4.1 ng/mL (2.7 to 4.1 μg/L) with a Tg antibody <0.4 U/mL(<0.4 kIU/L) over time (Fig. 1B). One year later, neck ultrasonography discovered a 2-cm midline mass with sonographic characteristics consistent with ectopic thyroglossal thyroid tissue. After removal of benign thyroid tissue in the thyroglossal duct, the Tg decreased to 2 to 3 ng/mL (2-3 μg/L). An I-123 WBS with SPECT/CT after recombinant human TSH stimulation was performed and demonstrated 4 sites of uptake in the thyroid bed consistent with residual thyroid tissue, but focal uptake in a 1-cm lytic lesion in the right frontal bone suggested a metastasis (Fig. 3A-D). The lesion was nontender and asymptomatic.

## Diagnostic Assessment

### Case 1

**Figure 2.**
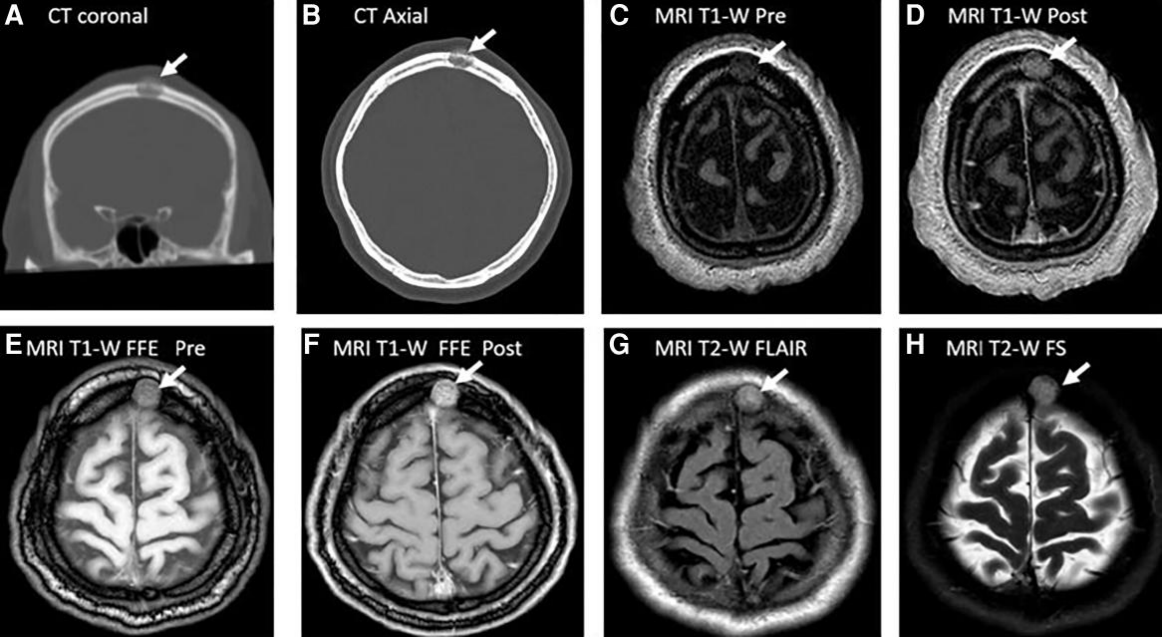
CT and MRI scans of a high-risk patient (case 1) with an intraosseous hemangioma (arrow). (A) CT scan without contrast, coronal image, with a 1.6 × 2 × 1.4-cm (TR × AP × CC) lytic lesion in the left superior frontal calvarium with interruption of the inner and out bone tables. (B) CT scan without contrast, axial image, showing the lytic lesion. (C) MRI scan, T1-weighted (T1-W) pregadolinium, axial image. (D) MRI scan, T1-weighted postgadolinium, axial image, showing an enhancing lesion with heterogeneous enhancement. (E) MRI scan, T1-weighted fast field echo (FFE) pregadolinium, axial image, showing a more obvious lesion with a fat suppressed signal. (F) MRI scan, T1-weighted FFE postgadolinium, axial image with improved visualization of the lesion with suppression of fat signal. (G) MRI scan, T2-weighted (T2-W) fluid attenuated inversion recovery (FLAIR), axial image, suppresses CSF signal showing a hyperintense lesion. (H) MRI scan, T2-weighted fat-suppressed (FS), axial image further improves lesion detection with suppression of the hyperintense fat signal.

A head CT scan showed a lytic lesion (Fig. 2A and B) with destruction of the endosteal cortical plate (inner bone table) and the periosteal cortical plate (outer bone table) of the skull. A brain MRI scan confirmed a 1.6 × 2 × 1.4-cm lytic lesion with soft-tissue extension beyond the outer bone table. The lesion was heterogeneous on pre-gadolinium contrast T1-weighted images (Fig. 2C), with enhancement after gadolinium infusion (Fig. 2D). The lesion was more obvious with specialized MRI sequences: fast field echo (FFE) (Fig. 2E and F) and T2-weighted images performed with fluid-attenuated inversion recovery (FLAIR) (Fig. 2G) and fat suppression (FS) (Fig. 2H). The destruction of the inner and outer bone tables, extracranial soft-tissue extension, and accelerating rise in Tg strongly suggested a cranial metastasis with the potential for invasion into the brain parenchyma. As a result, the lesion was resected but the pathology revealed an IH with no evidence of malignancy.

### Case 2

**Figure 3.**
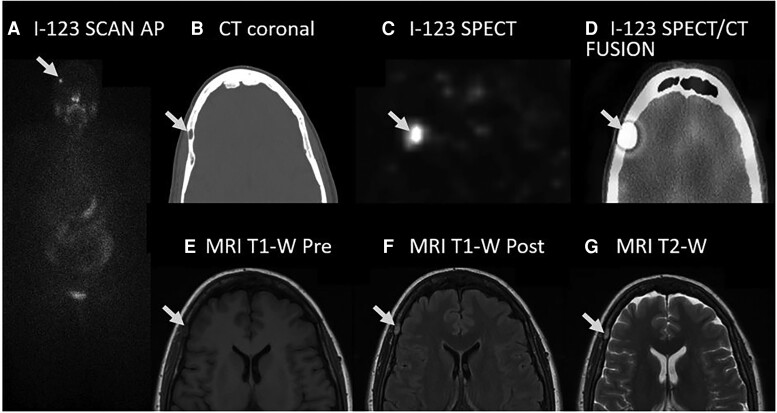
I-123 WBS scan with SPECT/CT imaging and MRI scan of low-risk patient with an interosseus hemangioma (arrow). (A) I-123 WBS anteroposterior (AP) planar image showing iodine trapping in the skull. (B) CT scan, coronal image, without contrast showing a 1.0 × 0.5-cm lytic lesion (arrow) in the right frontal bone. (C) I-123 SPECT scan, coronal image, showing iodine trapping in the right frontal bone. (D) I-123 SPECT/CT fusion scan, coronal image, showing iodine trapping in the lytic lesion (arrow). (E) MRI scan, coronal image, T1-weighted (T1-W) pregadolinium infusion showing hypodense lesion. (F) MRI scan, coronal image, T1-weighted coronal image postgadolinium infusion showing enhancement of the right frontal bone lesion. (G) MRI scan, coronal image, T2-weighted (T2-W) showing enhancement of the right frontal bone lesion.

This finding was stable on comparison to a CT scan performed 5 years earlier and a brain MRI scan performed 3 years earlier for headaches, when the lytic lesion had been regarded as characteristic of an IH. Review of the MRI scan shows the lesion was 1.0 × 0.5 cm and hypointense on T1-weighted images (Fig. 3E) that enhanced after gadolinium infusion (Fig. 3F) and hyperintense on T2-weighted images (Fig. 3G). Although the cranial lesion has not been resected, a benign IH is favored based on the lack of growth over 5 years, low-risk thyroid cancer staging, and typical IH MRI and CT scan characteristics.

## Treatment Outcome and Follow-up

### Case 1

The TSH-suppressed Tg continued to rise without change in the small lung nodules on CT scan, consistent with tumor progression likely in additional nonvisualized micrometastases in the lung.

### Case 2

No intervention was performed. A repet brain MRI 1 year later showed to change in the lytic lesion and her thyroglobulin level has remained low and stable.

## Discussion

Benign and malignant disease may develop in the cranium. Malignant lesions may be osteolytic, sclerotic, or mixed. Single osteolytic tumors are particularly worrisome for metastases from thyroid cancer. However, in this report, a single osteolytic lesion in the frontal bone of 2 patients with thyroid cancer was IH. IH are rare and account for fewer than 1% of all bone neoplasms [5-9]. When these IH are found in the cranium, they account for 0.2% of all bone tumors and 10% of benign skull tumors. In order of decreasing frequency, IH is found in the frontal bone, parietal bone, temporal bone, occipital bone, and the base of the skull [5, 7, 8]. The pathogenesis of IH is unknown, but these vascular bone tumors grow slowly and present as palpable hard masses sometimes with mild tenderness [5, 6, 9]. Growth of IH occurs by expansion of the outer bone table, with relative sparing of the inner bone table. These lesions may be asymptomatic and incidentally detected on imaging studies [7]. CT, PET, and MRI scans are commonly used to investigate IH [6, 7, 10]. IH do not always have typical radiologic features and should always be included in the differential diagnosis of malignant skull lesion [8, 10]. CT scans best elucidate bone changes, whereas MRI scans are more adept at evaluating the soft tissue. CT scan features of an IH include a well-circumscribed osteolytic lesion that typically expands through the outer bone table, with a “soap bubble,” “honeycomb,” or “sunburst” appearance that radiates from the center of the lesion [5]. In case 1, the CT images suggested destruction of both the inner and outer tables of the frontal bone and extension beyond bone suggestive of a metastasis, which led to the decision for surgical removal. The MRI features include variable signal intensity on both T1-weighted and T2-weighted imaging depending on the rate of blood flow through the dilated blood vessels and the amount of fat tissue in the lesion and surrounding bone marrow [7]. Bone metastases and IH both appear as nonhomogenously hypo- to iso-intense on T1-weighted and heterogeneously hyperintense on T2-weighted imaging [7, 8, 10], with enhancement of signal following gadolinium administration. On T2-weighted imaging, a bright signal also indicates slow flow or pooling of blood [7]. When IH are detected incidentally, the lesion is characterized on MRI only by T1-weighted images before and after gadolinium contrast administration and T2-weighted images. IH detection can be obscured by variable blood flow and fat in the lesion and the surrounding bone and bone marrow.

### Case 1

In case 1, additional MRI sequences were ordered to improve characterization of a skull lesion including FFE, FLAIR, and fat-suppressed images (Fig. 3C–H). Nuclear scans such as 99m-Technetium hydroxydiphosphonate (bone scan) are frequently used to identify metabolically active bone lesions. However, a bone scan may have radionuclide uptake in benign lesions such brown tumors associated with primary hyperparathyroidism. Furthermore, bone scans are frequently unremarkable in the setting of multiple myeloma and differentiated thyroid cancer. On F18-FDG PET scans, IH should have an absence of metabolic activity because of low glucose metabolism. Nevertheless, case reports describe false-positive hypermetabolic activity in IH that is presumably a function of relative stasis of blood with reduced washout of isotope [10]. Therefore, PET imaging does not have a strong role in distinguishing IH from a malignancy [10]. If imaging does not confirm a diagnosis, percutaneous skull biopsy is not usually recommended because of the vascular nature of bone with risks of bleeding and infection, especially if both tables of the bone are involved with the mass.

### Case 2

This case report highlights a benign bone pathology, IH, that can be mistaken for metastatic thyroid cancer when it presents as a lytic lesion on CT scan, radioiodine-persistent lesion on radioiodine WBS, enhancing destructive lesion with extracranial extension on MRI, and/or a hypermetabolic lesion on F18-FDG-PET scans. CT and MRI scans have complementary roles in helping determine the nature of cranial lesions. When a skull lesion is suspected, MRI sequences (FFE, FLAIR, and FS) may improve visualization by suppression of the signals from cerebrospinal fluid and lesional/cranial fat. Because no imaging studies are definitive for an IH, clinical decision-making needs to include consideration of the current risk of that patient for metastatic disease and surgical excision for pathological diagnosis to avoid unnecessary treatment of metastatic disease. Biopsy is not recommended because of the risk of bleeding and infection of the vascular tumor.

## Learning Points

- Intraosseous hemangiomas (IHs) are rare benign vascular tumors of the bone and, when occurring in the cranium, most often located in the frontal bone.
- Computed tomography and magnetic resonance imaging scans are complementary in the diagnosis of IH, but many of their characteristics are typical of metastatic tumors.
- Additional magnetic resonance imaging sequences will improve lesional detection by suppressing signal from cerebral spinal fluid (T2-weighted fluid-attenuated inversion recovery) and lesional/cranial fat (T1-weighted fast field echo and T2-weighted fat suppression).
- False-positive radioactive iodine whole body scans and F18-FDG positron emission tomography scans may occur because trapping of isotope in IH with slow blood clearance.
- Because no imaging studies are definitive for an IH, bone cranial lesions may warrant resection to establish a diagnosis, avoid potential brain invasion by a malignancy, or unnecessary radioiodine treatment if iodine avid.

## Contributors

All authors made contributions to authorship. H.K. was involved in the preparation of the manuscript. S.L.L., F.T.D., and D.M. were involved in the management of these patients and manuscript preparation. All authors approved the final draft.

## Data Availability

Original data generated and analyzed during this study are included in this published article.

## Abbreviations

CT: computed tomography
FFE: fast field echo
FLAIR: fluid-attenuated inversion recovery
FS: fat suppression
IH: intraosseous hemangioma
MRI: magnetic resonance imaging
PET: positron emission tomography
PTC: papillary thyroid cancer
SPECT: single photon emission computed tomography
Tg: thyroglobulin
WBS: whole body scan
